# Distinguishing linear and branched evolution given single-cell DNA sequencing data of tumors

**DOI:** 10.1186/s13015-021-00194-5

**Published:** 2021-07-06

**Authors:** Leah L. Weber, Mohammed El-Kebir

**Affiliations:** Department of Computer Science, University of Illinois at Urbana-Champaign, Urbana, IL 61801 USA

**Keywords:** Intra-tumor heterogeneity, Perfect phylogeny, Constraint programming, Single-cell DNA sequencing, Perfect phylogeny

## Abstract

**Background:**

Cancer arises from an evolutionary process where somatic mutations give rise to clonal expansions. Reconstructing this evolutionary process is useful for treatment decision-making as well as understanding evolutionary patterns across patients and cancer types. In particular, classifying a tumor’s evolutionary process as either linear or branched and understanding what cancer types and which patients have each of these trajectories could provide useful insights for both clinicians and researchers. While comprehensive cancer phylogeny inference from single-cell DNA sequencing data is challenging due to limitations with current sequencing technology and the complexity of the resulting problem, current data might provide sufficient signal to accurately classify a tumor’s evolutionary history as either linear or branched.

**Results:**

We introduce the Linear Perfect Phylogeny Flipping (LPPF) problem as a means of testing two alternative hypotheses for the pattern of evolution, which we prove to be NP-hard. We develop Phyolin, which uses constraint programming to solve the LPPF problem. Through both in silico experiments and real data application, we demonstrate the performance of our method, outperforming a competing machine learning approach.

**Conclusion:**

Phyolin is an accurate, easy to use and fast method for classifying an evolutionary trajectory as linear or branched given a tumor’s single-cell DNA sequencing data.

## Background

The clonal theory of cancer states that tumors arise from the accumulation of somatic mutations in a population of cells [1]. This process leads to a tumor comprised of heterogeneous *clones*—groups of cells with similar genotypes—or what is commonly referred to as intra-tumor heterogeneity. By performing bulk and/or single-cell DNA sequencing of a heterogeneous tumor biopsy, researchers and clinicians may infer reasonable models of this evolutionary process for important downstream analysis and clinical decision-making. Specifically, the evolution of a tumor is represented by a phylogeny, i.e. a rooted tree where the leaves of the tree represent the extant cells of the tumor, internal vertices represent ancestral tumor cells, and the root represents a normal cell. Due to trade-offs between the two data types, techniques for phylogeny inference have been developed for either bulk sequencing or single-cell data individually [2–6] as well as combined for joint inference [7–9]. Bulk sequencing data is less costly than single-cell data but results in a set of plausible phylogenies making it difficult to uniquely determine the true evolutionary history of a tumor [10, 11]. Conversely, single-cell data allows more precise reconstructions of a tumor’s evolutionary history but is subject to high rates of sequencing errors and is more expensive than bulk sequencing. In particular, single-cell sequencing has a high false negative rate, as much as 40% [12], implying that actual mutations present in a cell might not be indicated correctly in the resulting data. Doublets, where multiple cells are simultaneously sequenced as a single cell, are also a unique challenge of single-cell data. Less problematic are false positives, which indicate the presence of a mutation that is not present in a cell. While these rates are low (ranging from 0.0001 to 0.001 [12]) in comparison to false negative rates, it is important to account for false positives when inferring the evolutionary history of tumor.

One important open question is whether certain types or subtypes of cancers follow specific evolutionary patterns. Since tumors are typically biopsied at only a single point in time for reasons related to patient care, there does not yet exist sufficient longitudinal data to fully answer this question. However, it is believed that there are four high-level categories of tumor evolution: linear evolution, branched evolution, neutral evolution and punctuated evolution [13]. Linear and branched evolution are the focus of this work as neutral and punctuated evolution are special cases of these two high level categories. Linear evolution results when subsequent driver mutations develop a strong selective advantage and outcompete other clones during a clonal expansion [13]. By contrast, in branched evolution, a clone can diverge into separate lineages resulting in distinct branches and a tree-like model of evolution.

A useful first step in gaining insight into the evolutionary patterns of different cancer types is to compare the likelihood of the observed single-cell data under these two alternative hypotheses, linear and branched. Suppose we are given single-cell data in the form of a matrix where each row in the data is a cell and each column is a single-nucleotide variant (SNV), hereafter referred to as mutation. The entries in the data would then be either 1 or 0 indicating the presence or absence of a mutation in a particular cell. Suppose also that we assume a model of linear evolution and are given a false negative rate for the technology under which the single-cell sequencing was performed.

We could then determine the minimum number of changes from 0 to 1, indicating the entry was a false negative, such that the data is representative of a linear perfect phylogeny. This would then allow us to estimate the likelihood of the data under a hypothesis of linear evolution with false negative rate distribution in accordance with the sequencing technology. Under an alternative model of branched evolution, the number of flips required to obtain a linear perfect phylogeny and the associated false negative rate would follow a different distribution since many more flips would be required to not only correct actual false negatives but also to remove the implied branching.

Azer et al. [14] utilized a deep learning approach to decide if single-cell data indicates whether a tumor followed a linear or branched evolutionary process. Although their method is fast at prediction time and performs well on simulated data, it has not yet been proved whether the problem of identifying the minimum number of flips to obtain a linear perfect phylogeny is NP-hard. Additionally, the neural networks are trained on inputs of a fixed size. While padding could be used in predicting an input smaller than the fixed size [14], a new network would have to be trained if the input size is larger than the trained network. This drawback significantly reduces the speed advantage of such an approach as training of neural networks is a time consuming process.

**Fig. 1:**
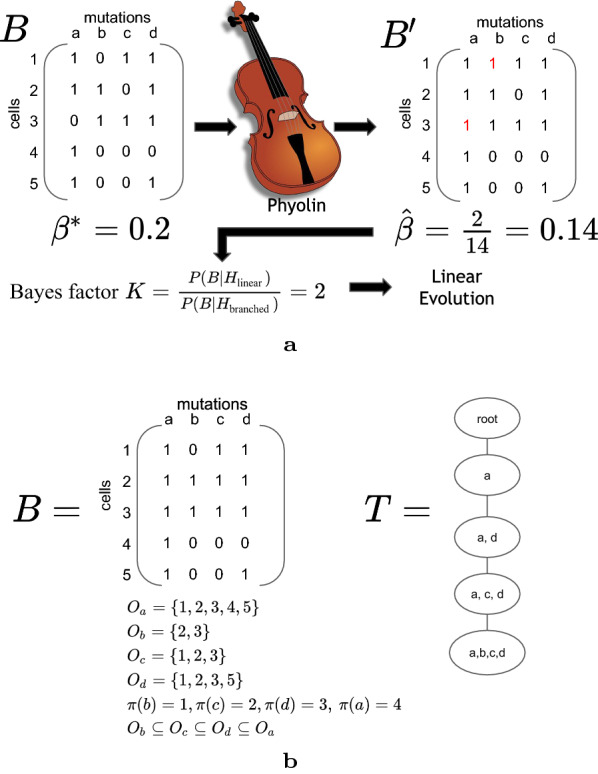
**a** A graphical depiction of Phyolin identifying a linear perfect phylogeny in single-cell DNA sequencing data when given a binary matrix *B* and a false negative rate $\beta ^*$. **b** An example of error free single-cell data that represents a linear perfect phylogeny and the equivalent clonal tree representation

Here, we prove that the problem of determining the minimum number of flips from 0 to 1 in single-cell data in order for the data to represent a linear phylogeny under the infinite sites model is NP-hard. We develop a method called Phyolin that makes use of constraint programming to find the minimum number of flips required to represent a linear perfect phylogeny. The outputted number of flips from Phyolin is then used to compute the strength of the evidence of the two alternatives of the pattern of evolution (Fig. 1a). We evaluate the performance of Phyolin on both simulated and real datasets, demonstrating that our method is an accurate and reasonably fast method for classifying an evolutionary trajectory as linear or branched.

## Preliminaries

Let *n* be the number of single cells sequenced and *m* be the number of unique mutations present in the *n* cells. In the following, after introducing the Linear Perfect Phylogeny Flipping problem, we describes two alternatives models of evolution.

### Problem statement

Under the infinite sites assumption (ISA), where each mutation *i* is gained exactly once and never subsequently lost, each sequenced cell corresponds to a leaf and we may infer a two-state perfect phylogeny using a polynomial time algorithm [15] where the binary character states encode the presence of mutation *j* in a cell *i*. We may equivalently represent a perfect phylogeny *T* as a binary matrix $B \in \{0,1\}^{n \times m}$ where $b_{ij} =1$ if cell *i* harbors mutation *j* and 0 otherwise. We provide the following definition [16] for convenience.

#### **Definition 1**

Given an *n* by *m* binary-character matrix *B* for *n* cells and *m* mutations, a *perfect phylogeny for* *B* is a rooted tree *T* with exactly *n* leaves provided that:

1. Each of the *n* cells labels exactly one leaf of *T*.
2. Each of the *m* mutations labels exactly one edge of *T*.
3. For any cell *p*, the mutations that label the edges along the unique path from the corresponding leaf to the root specify all of the mutations of *p* whose state is one.

Next, we formalize the notation of a set of cells that contain a mutation as the *one state*.

#### **Definition 2**

The *one state* $O_j$ of mutation *j* is the set of single cells *i* where $b_{ij} = 1$.

A perfect phylogeny *T* either depicts linear evolution or branched evolution. Intuitively, a matrix *B* represents linear evolution if there exists a total order of the set of *one states* with respect to the subset relation. Otherwise, we say perfect phylogeny *T* represents branched evolution. Also, we note that perfect phylogeny *T* is not necessarily bifurcating.

Utilizing the collection of *one states* for all *m* mutations, we determine if a given binary matrix *B* represents a linear perfect phylogeny as follows (Fig. 1b).

#### **Definition 3**

A binary matrix $B \in \{0,1\}^{n \times m}$ is a *linear perfect phylogeny* if there exists a permutation $\pi : [m] \rightarrow [m]$ such that $O_{\pi (1)} \subseteq \cdots \subseteq O_{\pi (m)}.$

However, single-cell sequencing is not error free and matrix *B* can fail to represent a linear perfect phylogeny even when it is representative of the true evolutionary process. False negatives, where a mutation that is present is not indicated as such, are particularly problematic with rates of up to 0.4 [12]. False positives, where absent mutations are indicated as present, are less of an issue in practice with rates less than 0.0005 for typically used whole-genome amplification strategies [17]. If the false positive error rate is known, we may estimate the expected number *z* of false positives in the observed data.

Given that false negatives are particularly prevalent, we would like to know how many false negatives would have had to occur in order for the inferred perfect phylogeny under the ISA to have a linear structure? This leads to the following problem statement.

#### **Problem 1**

(Linear Perfect Phylogeny Flipping (lppf)) Given a matrix $B \in \{0,1\}^{n \times m}$ and an integer *z*, find the minimum number of bit flips from 0 to 1 in *B* yielding a linear perfect phylogeny and the number of bit flips from 1 *to* 0 is at most *z*.

As we describe in “Methods”, upon obtaining the solution to the lppf, we utilize the total number of flips to distinguish whether the observed single-cell binary matrix *B* better supports linear or branched evolution.

## Complexity

Following [18], we prove that lppf is NP-hard by a reduction from the chain graph insertion problem, a known NP-complete problem [19].

### **Theorem 1**

lppf* is NP-hard.*

### *Proof*

We prove that lppf is NP-hard by considering a decision version *k*-lppf asking whether there exist *k* bit flips in input matrix *B* from 0 to 1 yielding a linear perfect phylogeny when the number *z* of the reverse flips (1 to 0) is 0. We claim that *k*-lppf is NP-complete by reduction from the chain graph insertion problem.

We begin by stating the definition of a chain graph and introduce the chain graph insertion problem.

### **Definition 4**

A bipartite graph $G = (X \cup Y, E)$ is a *chain graph* if there exists a permutation $\phi : \{1, \ldots , |Y|\} \rightarrow Y$ such that $\eta (\phi (1)) \subseteq \cdots \subseteq \eta (\phi (|Y|)$ where $\eta (v) =\{w \in X \mid (v,w) \in E\}$ is the set of adjacent nodes of *v*.

### **Problem 2**

(Chain Graph Insertion (cgi) [19]) Given a bipartite graph $G = (X \cup Y,E)$ and integer *k*, does there exist a chain graph $G^{\prime} = (X \cup Y,E^{\prime})$ such that $E \subseteq E^{\prime}$ and $|E| + k = |E^{\prime}|$?

*k*-lppf $\in$ NP because given a certificate (set of *k* flips from 0 to 1) to *k*-lppf, we could order the columns by increasing cardinality of the resulting one state sets and then check if that permutation satisfies the definition of a linear perfect phylogeny. We will now show that cgi $\le _p$ *k*-lppf.

**Fig. 2:**
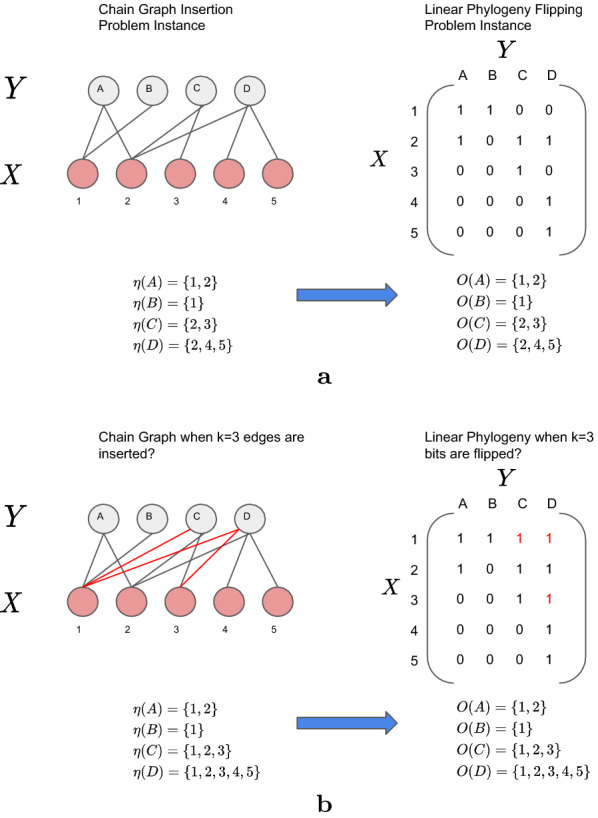
Chain graph insertion problem reduction. **a** Polynomial time reduction of the cgi to lppf. **b** An equivalent solution of the cgi and lppf when $k=3$

Starting from an instance $(G=(X\cup Y,E),k)$ of cgi, we construct an instance *B* of *k*-lppf in the following manner. Binary matrix *B* has |*X*| rows and |*Y*| columns, and its entries are directly obtained from the edge set *E* of *G*: For each $v \in Y$, we set $b_{iv} = 1$ if *i* is a neighbor of *v* for all $i \in X$ and let $b_{iv} = 0$ otherwise. This can be done in polynomial time. Figure 2a demonstrates the polynomial time reduction. We claim that *k* edge insertions suffice to obtain a chain graph from *G* if and only if *B* represents a linear perfect phylogeny when *k* bits are flipped from 0 to 1 and no bits are flipped from 1 to 0 (i.e. $z=0$).

$(\Longrightarrow )$ Suppose $(G,k) \in$ cgi. Then there exists an edge set $D \subseteq \{(u,v) \mid u \in X, v \in Y\} \setminus E$ such that $|D| = k$ and $H= (X \cup Y, E \cup D)$ is a chain graph. Then by definition of a chain graph, there exists an permutation $\phi : \{1, \ldots , |Y|\} \rightarrow Y$ such that $\eta (\phi (1)) \subseteq \cdots \subseteq \eta (\phi (|Y|)$. It is easy to see that by construction, $\eta (v) = O_v$, for all $v \in Y$. Since $\phi$ exists, a permutation $\pi$ of the one states also exists. Therefore, *B* represents a linear perfect phylogeny after flipping the 0-bits encoded in set *D*.

$(\Longleftarrow )$ Suppose $(B,k) \in$ *k*-lppf. Then there exists a set *F* of positions (*i*, *j*) where $b_{ij} =0$ to $b_{ij} = 1$ such that $|F| =k$. Let $B^*$ be the resulting matrix after each flip at position $(i,j) \in F$ is made. Then $B^*$ represents a linear perfect phylogeny and there exists a permutation $\pi : \{1, \ldots , m\} \rightarrow [m]$ such that $O_{\pi (1)} \cdots \subseteq O_{\pi (|m|)}$. Using the equivalence between one-states and neighbors, $H = (X \cup Y,E)$ can be constructed from $B^*$ in the following manner. First, create the set $X =[n]$ from the rows of $B^*$ and the set $Y= [m]$ from the columns of $B^*$. Then, create the set *E* of edges as $\{ (x,y) \in X \times Y \mid b^*_{xy}=1$}. By construction, $H = (X \cup Y, E)$ is a chain graph. $\square$

## Methods

The following section describes Phyolin, which solves the lppf problem and computes the Bayes factor for distinguishing linear and branched evolution. We implemented Phyolin in C++ utilizing IBM ILOG CP OPTIMIZER^1^. Phyolin is publicly available at https://github.com/elkebir-group/phyolin.

### Model

To solve the lppf, we formulate a constraint optimization problem (COP) [20]. A COP is a constraint satisfaction problem (CSP) with an objective function that specifies which feasible solutions are preferred based on an optimization criteria. A CSP is defined by a tuple $({\mathcal {X}},{\mathcal {D}}, {\mathcal {C}})$, where ${\mathcal {X}} = \{x_1, \ldots ,x_n\}$ is the set of decision variables, ${\mathcal {D}} = \{d_1, \ldots d_n\}$ is the set of domains for ${\mathcal {X}}$ and ${\mathcal {C}}$ is a set of constraints that must be satisfied. A solution $a \in {\mathcal {A}}({\mathcal {X}},{\mathcal {D}},{\mathcal {C}})$ to a CSP is an assignment of values $\{x_1 \mapsto v_1, \ldots , x_n \mapsto v_n\}$ such that $v_i \in d_i$ for all $i \in [n]$ and all constraints *C* are satisfied. To facilitate an objective function $f: {\mathcal {A}}({\mathcal {X}},{\mathcal {D}},{\mathcal {C}}) \rightarrow {\mathbb {R}}$, an initial assignment ${\hat{a}} \in {\mathcal {A}}({\mathcal {X}},{\mathcal {D}},{\mathcal {C}})$ is found. Then, a preference constraint is added to *C*, such that $f(a) \le f({\hat{a}})$ for a minimization problem or $f(a) \ge f({\hat{a}})$ for a maximization problem. The search is continued and the preference constraint updated each time a feasible assignment ${\hat{a}}$ is found until no more feasible assignments exist. When this occurs, the assignment ${\hat{a}}$ is returned and $f({\hat{a}})$ is the objective value. We note that in problems where multiple optimal solutions exist, it is possible to return all such valid solutions. But even though multiple solutions may exist, our focus is on assessing the plausibility of the null hypothesis of linear evolution. Therefore, it is sufficient to consider any optimal solution even if the respective assignments yield different linear perfect phylogenies.

First, we describe the set ${\mathcal {X}}$ of decision variables and the associated domains ${\mathcal {D}}$ used in the formulation. The set ${\mathcal {X}}$ contains the variables $\mathbf{x}$ and $\mathbf{c}$. Intuitively, the values taken by $\mathbf{x}$ represent a binary matrix $B^{\prime}$ used to represent a linear perfect phylogeny after flipping. More formally, given a set *n* of cells and a set *m* of mutations, let $x_{ij}$ = 1 if cell *i* has mutation *j* in the linear perfect phylogeny $B^{\prime}$ after flipping and 0 otherwise for each cell $i \in [n]$, and mutation $j \in [m]$. Then, ${\mathcal {D}}(x_{ij}) = \{0,1\}$, for all $i \in [n], j \in [m]$. The variables $\mathbf{c}$, are used to define a permutation of the columns in $B^{\prime}$, such that after flipping is completed, $B^{\prime}$ will adhere to the definition of a linear perfect phylogeny. Recall that in order to represent a linear perfect phylogeny, there must exist permutation $\pi : [m] \rightarrow [m]$ such that $O_{\pi (1)} \subseteq O_{\pi (2)} \subseteq \cdots \subseteq O_{\pi (m)}$. Let $c_j = \pi (j)$ for all $j \in [m]$. Then ${\mathcal {D}}(c_j) = [m]$ for all $j \in [m]$.

Since, our goal is to find the linear perfect phylogeny that requires as few flips as possible, that is minimizing the number of false negatives we infer, we define an objective function that minimizes the number of flips from 0 to 1.

1 $$\min \sum _{i=1}^n \sum _{j=1}^m \mathbb {1}(b_{ij}=0, x_{ij} =1).$$

The set ${\mathcal {C}}$ ensures that the outputted binary matrix $B^{\prime}$ meets the conditions of representing a linear perfect phylogeny and also that the number of false positives, or flips from 1 to 0, is bounded above by $z^*$. The set ${\mathcal {C}}$ consists of the following three constraints.

2 $$\textsc {ALLDIFFERENT}(c),$$

3 $$\sum _{i=1}^{n}\sum _{j=1}^m\mathbb {1}(b_{ij}=1, x_{ij} =0) \le z^*$$

4 $$(c_k < c_j) \Rightarrow (x_{ij} \le x_{ik}) \quad \forall k,j \in [m], \forall i \in [n].$$

Equation (2) is a global constraint that ensures that every mutation is assigned a unique ordering in the permutation. Equation (3) ensures that the number of false positives or flips from 1 to 0 is bounded above by the number of allowable false positives $z^*$, as determined by a given false positive rate $\alpha$. Finally, Eq. (4) ensures the defining property of a linear perfect phylogeny is met by ensuring that if $\pi (k)$ is less than $\pi (j)$ then it must hold that $O_k \subseteq O_j$ for all $k,j \in [m]$.

### Two alternative hypotheses for the pattern of evolution

We have two competing hypotheses to explain the observed data *B*: (i) linear evolution ($H_{\text {linear}}$) and (ii) branched evolution ($H_{\text {branched}}$). The solution to the lppf is the total number *y* of flips from 0 to 1 in order to represent linear evolution. In addition, we obtain a matrix $B^{\prime}$ containing the resultant bits after flipping. Let *N* be the total number of ones in $B^{\prime}$ and ${\hat{\beta }} = y/N$ represent the fraction of false negatives in the solution to the lppf. We then hypothesize *y* was drawn from one of two different beta-binomial distributions.

Under the null hypothesis $H_{\text {linear}}$ of linear evolution, we have

5 $$p_\text {linear} \sim \text {beta}(\mu _{\text {linear}}, s_{\text {linear}}),$$

6 $$y \sim \text {binomial}(N, p_\text {linear}),$$

where the mean $\mu _\text {linear}$ equals the expected false negative rate $\beta ^*$ and $s_{\text {linear}}$ is the beta-binomial precision parameter (controlling dispersion) of the used sequencing technology.

In the worst case, every 0 can be flipped to a 1. This results in a binary matrix with all values equal to 1, suggesting a linear perfect phylogeny with a single clone harboring all of the mutations. The number of flips required to achieve such a solution may be implausible under a model of linear evolution, which seeks to characterize the true distribution of false negatives for the sequencing. Thus, we consider an alternative hypothesis that characterizes the distribution of flips when a branched phylogeny is forced to be linear.

Under the alternative hypothesis $H_\text {branched}$ of branched evolution, $H_{\phi }$, we have

7 $$p_{\text {branched}} \sim \text {beta}( \mu _{\text {branched}}, s_{\text {branched}}),$$

8 $$y \sim \text {binomial}(N, p_\text {branched}),$$

where $\mu _\text {branched}$ and $s_\text {branched}$ parameterize a distribution that results from converting data generated under branched evolution into one that represents a linear perfect phylogeny. In other words, we expect the estimated false negative rate ${\hat{\beta }}_\phi = y/N$ to follow a different distribution from that of the false negative rate $\beta ^*$ of the sequencing technology since the number of flips for branched evolution will be greater for data generated under linear evolution.

While $\mu _\text {linear}$ and $s_\text {linear}$ are typically known, parameters $\mu _\text {branched}$ and $s_\text {branched}$ are more challenging to estimate. One approach is to generate simulated data under branched evolution and the given sequencing profile and utilize ${\hat{\beta }}_\phi$ to fit this distribution. As this is time consuming in practice, we offer two heuristics. First, given some hard threshold $\beta ^*$, which could be based on knowledge of the system estimated false negative rate or conservatively set at 0.4 [12], we reject $H_{\text {linear}}$ that the phylogeny is linear whenever $\hat{\beta } > \beta ^*$. Unlike the Bayes factor, a hard threshold does not provide any evidence in support of $H_{\text {linear}}$, only allowing us to conclude the pattern of evolution is branched. Second, as a more robust alternative, one can establish a hypothetical threshold beta-binomial distribution with parameters $\mu _\text {branched}$ and $s_\text {branched}$ representing an implausible distribution for the false negative rate of the sequencing technology.

### Bayes factor

To determine the hypothesis that best explains the observed data *B*, we utilize the Bayes factor. The Bayes factor

9 $$K= \frac{P(B \mid H_{\text {linear}})}{P(B \mid H_{\text {branched}})}$$

quantifies the likelihood ratio of the two computing hypotheses ($H_{\text {linear}}$ and $H_{\text {branched}}$) for the evolutionary pattern of the observed binary matrix *B*. A Bayes factor $K > 1$ is evidence in support of $H_{\text {linear}}$ and $K < 1$ is evidence in support of $H_{\text {branched}}$. Furthermore, Bayes factor *K* expresses how strongly the observed data supports one of the two alternative hypotheses.

Given two shape parameters $\theta _1$ and $\theta _2$, the likelihood of the beta-binomial distribution modeling each of our two hypotheses is defined as

10 $$f(y \mid , N \theta _1, \theta _2) = \left( {\begin{array}{c}N\\ y\end{array}}\right) \frac{{\mathcal {B}}(\theta _1 + y, N - y + \theta _2)}{{\mathcal {B}}(\theta _1, \theta _2)},$$

where ${\mathcal {B}}$ is the beta function.

We parameterize the statistical distribution of our hypotheses in terms of $\theta _1$ and $\theta _2$ via the conversion

11 $$\theta _{i,1}= \mu _i s_i,$$

12 $$\theta _{i,2}= s_i - \theta _{i1},$$

for $i \in \{\text {linear}, \text {branched}\}$. Finally, substituting Eq. (10) into Eq. (9) for both hypotheses $H_{\text {linear}}$ and $H_{\text {branched}}$ yields Bayes factor *K* equal to

13 $$\frac{{\mathcal {B}}(\theta _{\text {linear},1} + y, \theta _{\text {linear},2} + N -y){\mathcal {B}}(\theta _{\text {branched},1}, \theta _{\text {branched},2})}{{\mathcal {B}}(\theta _{\text {branched},1} + y, \theta _{\text {branched},2} + N -y){\mathcal {B}}(\theta _{\text {linear},1}, \theta _{\text {linear},2})}.$$

## Results

In order to evaluate Phyolin, we perform in silico experiments as well as run Phyolin on real data. First, we seek to evaluate the performance of Phyolin when the simulated data closely approximates a recently published high throughput single-cell DNA sequencing study of an acute myeloid leukemia (AML) cohort [21]. Second, we establish the robustness of Phyolin to false positives and doublets. Third, we describe the application of Phyolin to patients with childhood acute lymphoblastic leukemia [22]. Finally, we apply Phyolin to the AML cohort [21] that inspired the initial set of simulations. All experiments were conducted on a server with Intel Xeon Gold 5120 dual CPUs with 14 cores each at 2.20 GHz and 512 GB RAM.

### Simulations approximating an acute myeloid leukemia cohort

In a recent study, Morita et al. [21] performed DNA single-cell sequencing on a cohort of 123 patients with acute myeloid leukemia (AML) and inferred the evolutionary tree of each patient using SCITE [2]. Utilizing high-throughput sequencing resulted in a median of 7584 cells sequenced per patient [21]. AML is a cancer type where both linear and branching evolutionary patterns are suspected [21]. As a result, the set of trees published in Ref. [21] were a mix of linear and branched patterns. We design a simulation study to approximate a subset of patients in this cohort but where the ground truth evolutionary pattern is known. We use the published trees and clonal prevalences in conjunction with estimated per false negative rate $\beta = 0.05$ for the sequencing technology to generate simulated matrices *B* for a subset of 12 AML patients [21]. Specifically, the prevalence of clone *i* is the number of cells in the sample mapped to clone *i* divided by the total number of cells in the sample. We consider six patients with linear trees and mutations ranging from 3 to 5 and six patients with branched trees and mutations ranging from 3 to 7. A total of 10 replications were performed per simulated patient. Table 1 shows a summary of the patients selected for inclusion in the simulation study.

**Table 1:** Simulation study based on characteristics of a published AML cohort [21]

Patient	Pattern	# Mutations *m*	# Cells *n*	Median flips	Phyolin median flips	Median $\hat{\beta }$	Med prob.
AML-2	Linear	5	7931	1826	1039	0.037	0.70
AML-8	Linear	3	4675	759	294	0.029	0.42
AML-10	Linear	4	8729	1427	584	0.030	0.56
AML-33	Linear	3	8120	1091	350	0.027	0.41
AML-47	Linear	3	6491	1135	488	0.032	0.42
AML-58	Linear	3	8170	1280	472	0.029	0.40
AML-53	Branched	3	8013	544	2220	0.44	0.39
AML-62	Branched	6	4027	726.5	2299	0.19	0.58
AML-63	Branched	4	8347	1238.5	1432	0.084	0.44
AML-67	Branched	7	6024	1061.5	6440	0.31	0.71
AML-69	Branched	3	7462	651.5	1037	0.16	0.29
AML-74	Branched	5	9279	1020	2122	0.17	0.38

Shown is the patient identifier, the published evolutionary pattern of the tree, the number of mutations, the total cells sequenced [21], the median number of simulated false negatives over 10 replications, Phyolin estimated number of false negatives over 10 replications, the median $\hat{\beta }$ over 10 replications, and the median probability of a linear perfect phylogeny as determined by the comparison deep learning method [14]

To utilize the Bayes factor, we require knowledge of the parameters of the prior distribution, $\mu _\text {branched}$ and $s_\text {branched}$, under the branched evolution hypothesis $H_{\text {branched}}$. Since these are challenging to estimate, we demonstrate the first heuristic of classification utilizing a strict threshold at true false negative rate $\beta ^*= 0.05$ and the second heuristic of applying Bayes factor on a theoretical distribution with $\mu _\text {branched} = 0.15$ and $s_\text {branched}=10$. For the linear evolution hypothesis $H_{\text {linear}}$, we utilize $\mu _{\text {linear}} = 0.05$, with precision $s_\text {linear} = 10$, matching known values for this sequencing platform [21].

**Fig. 3:**
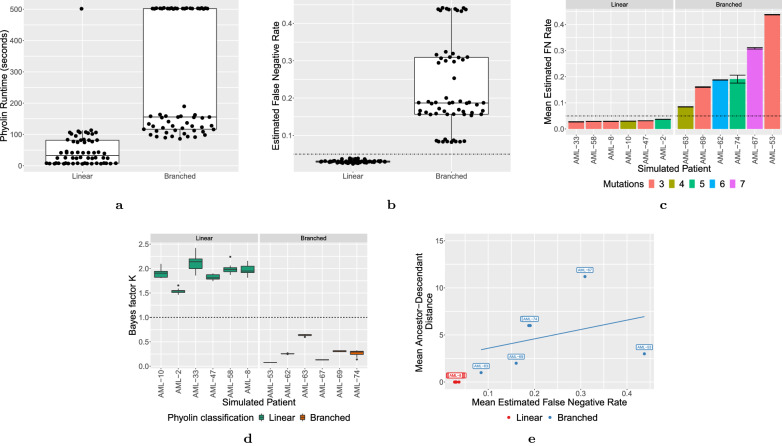
Phyolin results of the in silico experiments on a simulated cohort of patients with AML. **a** A comparison of Phyolin runtimes in seconds between instances with different evolutionary patterns. **b** A comparison of the distribution of $\hat{\beta }$ between simulated linear and branched topologies over 10 replications. **c** The mean value of $\hat{\beta }$ for each patient along with the standard error. **d** The distribution of Bayes factor *K* for each simulated patient separated by the true evolutionary pattern. The horizontal line at $K=1$ represents the classification criteria ($K > 1$ is classified as linear). **e** Relationship between estimated false negative rate and the ancestor–descendant distance. Each point represents the mean value over 10 replications and is labeled by the numerical patient identifier. A linear trend line is shown with a $95\%$ confidence interval

We set an upper limit on runtime of Phyolin at 500 s with 80% of the replications returning an optimal solution in under the time limit. We chose the 500 s time limit to facilitate timely analysis of the input data. Figure 3a shows the distribution of runtime by the simulated evolutionary pattern. Linear patterns resulted in a median runtime of 33 s (IQR: 8–82 s) and branched patterns resulted in a median of 156 s (IQR: 117–502 s). The median input size (cells $\times$ mutations) of the linear patterns was 24,435 and 28,775 for branched patterns. The largest input was for AML-74 with 9279 cells by 5 mutations and no replications completed within the time limit. Only 1 replication with a linear pattern did not complete within the time limit. This implies that optimal solutions are found much faster when the true pattern is linear.

Figure 3b compares the distribution of the estimated false negative rate $\hat{\beta }$ for the patients with linear versus branched published trees over all 10 replications. The simulated system false negative rate was $5\%$ and is shown as a dashed line where relevant. From Fig. 3b, we note a significant difference in the distributions between linear and branched instances. Additionally, the median of the linear perfect phylogeny patients is 0.03 (IQR: 0.028–0.032) and every linear replication is less than $\beta ^*=0.05$ while the median of the branched perfect phylogeny patients is 0.19 (IQR: 0.16–0.31) and every branched replication is greater than $\beta ^*=0.05$.

Figure 3c shows the mean $\hat{\beta }$ and standard error for each simulated patient over the 10 replications. The number of mutations are also shown in order to investigate if increasing number of mutations increases $\hat{\beta }$. Although there appears to be some effect when increasing the number of mutations, it does not strictly hold. However, utilizing a strict threshold of $\beta ^*=0.05$ results in perfect classification of the topology for all patients and all replications.

Figure 3d compares the difference between Bayes factor *K* for patients with simulated linear and branched perfect phylogenies. Furthermore, without the presence of other sequencing errors, classification via a threshold $\beta ^*=0.05$ and Bayes factor *K* are in agreement on all simulation replications. Additionally, Bayes factor *K* provides a more robust interpretation of the evidence for the two alternative hypotheses. For example, the median Bayes factor *K* for AML-63 is 0.65, which is maximum among all patients with simulated branching. AML-63 also had the lowest estimated median estimated false negative rate (${\hat{\beta }} = 0.084)$.

Since the number of mutations does not necessarily impact the estimated false negative rate $\hat{\beta }$, another consideration is whether or not $\hat{\beta }$ increases with the amount of branching. To this end, we compare the ancestor–descendant distance between the simulated true tree $B^*$ and the inferred linear tree $B^{\prime}$. A mutation *x* is an *ancestor* of mutation *y* if *x* occurs on the path from the root to *y*, in which case *y* is said to be a descendant of *x*. *Ancestor–descendant* (AD) *distance* is defined as the size of the symmetric difference between the sets of ordered pairs of characters, or ancestor–descendant pairs, introduced on distinct edges of perfect phylogenies $B^*$ and $B^{\prime}$. A higher AD distance implies a greater degree of branching in the true tree. For example AML-63 has only one branch and AML-67 has three distinct branching events. Figure 3e shows the relationship between the mean estimated false negative rate $\hat{\beta }$ and the mean AD distance per simulated patient over 10 replications. The AD distance is 0 for all patients with a simulated linear perfect phylogeny. This means that Phyolin correctly infers the true tree when it is linear. In particular, for the branched instances, there is evidence of a correlation between the estimated false negative rate $\hat{\beta }$ and the degree of branching as captured by the AD distance (Fig. 3e).

Azer et al.’s [14] deep learning method for classifying topology is the most similar method for comparison with Phyolin. Therefore, we retrained this deep neural network to support our input size of 9300 cells and 7 mutations. We used a default hidden layer size of 100 and drop-out rate of 0.9, 5000 training examples and 500 epochs. We did not modify any other hyperparameters. The input size was selected so that only one network needed to be trained for all in silico experiments and we used padding for any instances where $n < 9300$ or $m < 7$. After 200 epochs the best validation accuracy was $64.1\%$ and completed in 2168 s (36.1 min). After 500 epochs, the best validation accuracy was $64.8\%$ and completed in 3997 s (66.6 min). This suggests that further learning was unlikely. We report the probability that the phylogeny is linear on the same simulation instances when evaluated with the trained model.

**Fig. 4:**
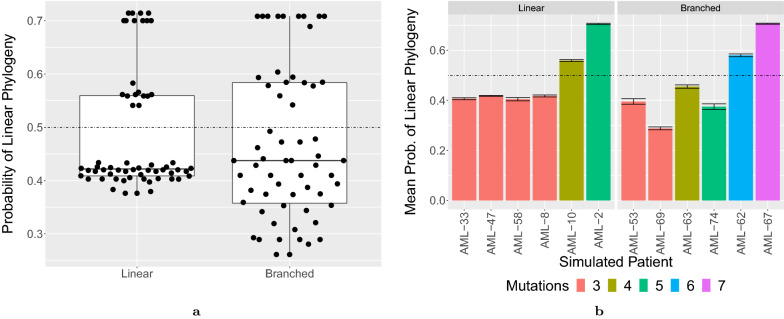
Results of the deep learning approach [14] applied to the in silico experiments on a simulated cohort of patients with AML. **a** A comparison of the distribution of the probability of a linear perfect phylogeny between simulated linear and branched topologies over 10 replications. **d** The mean value of the probability of a linear perfect phylogeny for each patient along with the standard error. A horizontal line indicates the threshold probability (0.5) used to classify an input as linear.

Table 1 shows the median probability that the phylogeny is linear over the 10 replications per simulated patient. We use a cutoff of 0.5 as the threshold for classifying a topology as linear. Figure 4a shows the distribution of the probabilities over all patient replications by ground truth topology. Classification accuracy was $100\%$ for 6 of the 12 simulated patients (Linear: AML-2, AML-10, Branched: AML-53, AML-63, AML-69 and AML-74) and $0\%$ for the remaining 6 simulated patients. Figure 4b shows mean estimated probability per patient and standard error for the 10 replications. The predicted probability tends to increase as the number of mutations increases.

In summary, the simulated AML cohort results show that, in contrast to the deep learning approach [14], Phyolin correctly and quickly classifies large instances as linear with a strict threshold $\beta ^*$ set at the system estimated false negative rate. Furthermore, as the amount of branching increases, the estimated ${\hat{\beta }}$ tends to increase. Thus, the greater the difference between $\hat{\beta }$ and $\beta ^*$, the more confident we can be in rejecting the hypothesis of linear evolution in favor of the alternative hypothesis of branched evolution.

### Robustness to false positives and doublets

While the previous simulation study focused only on false negatives, here we examine the robustness of Phyolin to the presence of false positives and doublets. In addition, we demonstrate the technique of estimating the parameters of branched evolution hypothesis $H_{\text {branched}}$. First, we generated a set of matrices representing a perfect phylogeny with 500 cells and mutations $m \in \{10,25,50\}$ using the $\texttt {ms}$ simulator [23]. We then introduced errors to each simulated matrix *B* with false negative probability $\beta \in \{0.05, 0.15, 0.25\}$, false positive probability $\alpha \in \{0.001, 0.01\}$ and doublet probability $\delta \in \{0.0, 0.2\}$. Note that $z = \lceil \alpha \sum _{i=1}^n\sum _{j=1}^m b_{ij}\rceil$ for each simulation instance. We performed 5 replications for each combination of conditions and each type of evolution, linear or branched, for a total of 360 instances. To account for the added complexity of false positives and doublets, we allowed each Phyolin instance to run for a maximum of 900 s. Independently, we created an additional set of experiments with only branched evolution with the same parameters as the simulation. We utilized this set of experiments to estimate $\mu _\text {branching}$ and $s_\text {branching}$ from the Phyolin outputted false negative probability $\beta _\text {branching}$ under branched evolution separately for each $m \in \{10, 25, 50\}$. Using these parameters, we determined the category of evolution as linear or branched in accordance with the Bayes factor *K*.

**Fig. 5:**
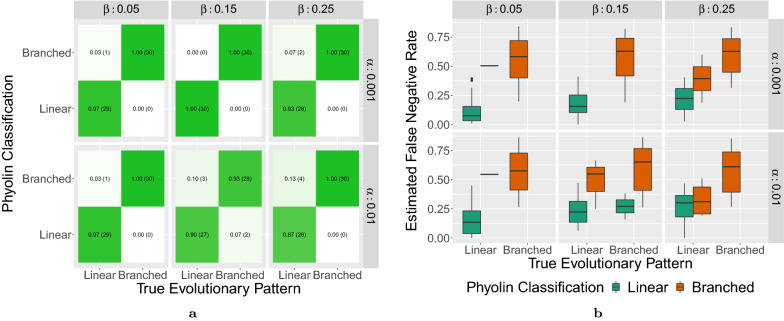
Phyolin demonstrates robustness to false positives and doublets. **a** The confusion matrix for Phyolin classifications versus the true evolutionary pattern annotated by the proportion (total) of instances. **b** The distribution of Phyolin estimated false negative rates by true evolutionary pattern with colors depicting the Phyolin classification at simulated false negative rates $\beta \in \{0.05, 0.15, 0.25\}$, simulated false positive rates $\alpha \in \{0.01, 0.001\}$ and for double rates $\delta \in \{0.0, 0.2\}$

We found Phyolin to be robust to both the presence of false positives and doublets with an overall accuracy of $97\%$ (Fig. 5a). For doublet probability $\delta =0.0$, 85 out of 90 branched instances were correctly classified with 5 instances incorrectly classified as linear (median Bayes factor $K=0.94$). A total of 87 out of 90 linear instances were correctly classified with 3 instances incorrectly classified as branched (median Bayes factor $K=0.005$). Paradoxically, for doublet probability $\delta =0.2$ performance improves with only two linear and two branched evolutionary patterns incorrectly classified. This is explained by the fact that when the true evolutionary pattern is branched, a doublet comprised of cells on distinct branches of a tree will result in fewer cases of branching that must be flipped by Phyolin. Therefore, when high doublet rates are suspected, experiments believed to be doublets should be removed from the data prior to utilizing Phyolin. In addition, we observe a significant difference in the Phyolin estimated false negative rate ${\hat{\beta }}$ between simulated instances with linear (median: 0.18) and branched (median 0.61) evolution (Fig. 5b).

### Real data of childhood acute lympoblastic leukemia patients

Gawad et al. [22] performed single-cell DNA sequencing on a cohort of six patients with childhood acute lymphoblastic leukemia (ALL). As a subtype of leukemia, ALL is also postulated to follow both linear and branched trajectories [24]. We evaluate Phyolin on two of the six patients in this cohort: Patient 2 and Patient 6 (Table 2). The input size for Patient 2 was 115 cells by 16 mutations with an estimated false negative rate $\beta^*=0.18$ [22]. Two independent, previous analyses of the sequencing data of Patient 2 suggested a branched topology [7, 25]. For Patient 2, Phyolin estimated a false negative rate of 0.36, which is much greater than the previously estimated rate of 0.18 [22]. Moreover, the Bayes factor $K=0.26$ implies accepting the branched evolution $H_{\text {branched}}$ hypothesis. This concurs with the branched trees published in [7, 25].

**Table 2:** Summary of Phyolin analysis of two patients with ALL

Patient	Cells sequenced [22]	Mutations	Phyolin flips	$\hat{\beta }$	$\beta ^*$ [22]	*K*
Patient 2	115	16	403	0.36	0.18	0.3
Patient 6	146	10	191	0.15	0.18	14.0

Shown is the patient identifier, the number of cells sequenced [22], the number of mutations, the number of flips performed by Phyolin, the estimated false negative rate ${\hat{\beta }}$, the false negative rate threshold $\beta ^*$ [26] that was estimated for the sequencing technology and Bayes factor *K*

In addition, we consider Patient 6 because this patient was analyzed by the deep learning method [14]. The input size for Patient 6 was 146 cells by 10 mutations with an estimated false negative rate $\beta^*=0.18$. For Patient 6, Phyolin estimated a false negative rate of 0.15, which is less than the published false negative rate $\beta ^*=0.18$ [22]. Bayes factor $K=14.0$ substantially supports the linear evolution hypothesis $H_{\text {linear}}$. The comparison deep learning approach [14] also concluded that the phylogeny was linear. Table 2 summarizes results obtained by Phyolin.

### Application to an acute myeloid leukemia cohort

We assess the performance of Phyolin by applying our method to single-cell DNA sequencing data for 9 of the 12 patients included in our simulation study. We exclude 3 patients (AML-10, AML-33, AML-58) with greater than 0.1 fraction of data missing. In addition, we analyze an additional 5 patients with trees highlighted in the original study [21] for a total of 14 patients (9 branched, 5 linear). To obtain the input matrices $B^{\prime}$, we extract variant and total read counts from the loom files generated via the Mission Bio Tapestri pipeline. To discretize the read counts, we use the binomial exact test with null error rate 0.001 and p-value $10^{-6}$. Sites with 0 total reads are labeled as missing data and ignored by Phyolin.

**Fig. 6:**
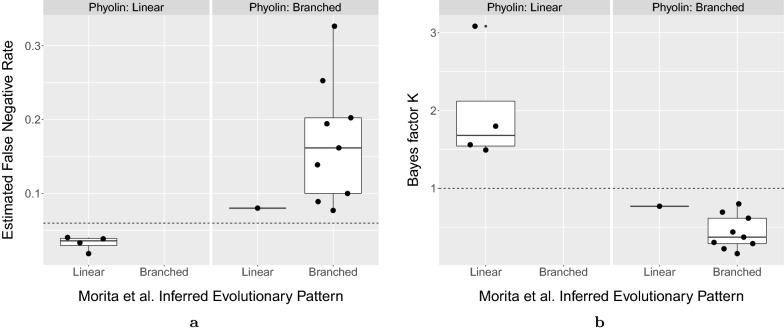
Phyolin concurs with Morita et al. [21, 27] on the evolutionary pattern on a cohort patients with acute myeloid leukemia. **a** The distribution of Phyolin estimated false negative rates by evolutionary pattern reported by Morita et al. and by Phyolin classification with published false negative rate $\beta ^*=0.06$ (horizontal line) and false positive $\alpha =0.01$ [21]. **b** The distribution of the Bayes factor *K* by evolutionary pattern reported by Morita et al. and by Phyolin classification with published false negative rate $\beta ^*=0.06$ and false positive $\alpha =0.01$ [21]. The horizontal line at $K=1$ depicts the threshold for classification

Given that the median estimate false negative rate for the cohort was $\beta =0.058$, we utilize prior parameters mean $\mu _\text {linear} = 0.058$ and precision $s_\text {linear}=10$ for our linear evolution hypothesis $H_{\text {linear}}$. We parameterize the branched evolution hypothesis $H_{\text {branched}}$ with $\mu _\text {branched} = 0.22$ and $s_\text {branched}=11.8$ guided by our simulation study. Phyolin’s classification of the evolutionary pattern corresponded to the reported trees [21] in 12 out of the 13 instances. Fig. 6a demonstrates a significant difference between the Phyolin estimated false negative rate for linear (median: 0.04) and branched evolutionary (median: 0.16) as determined by Morita et al. [21]. Furthermore, the Bayes factor *K* (Fig. 6b) similarly separates the two classes of evolutionary patterns as Morita et al. [21, 27]. Only patient AML-02 differed with Phyolin classifying the evolutionary pattern as branched (${\hat{\beta }}= 0.08$). However, in the initial bioRxiv supplement [27], Morita et al. reported 4 trees for each patient by running SCITE in different modes. We note that 2 trees were branched while 2 were linear. Thus, the Bayes factor $K=0.77$, helps contextualize the ambiguity surrounding the true evolutionary trajectory for this patient.

## Conclusions

In this work, we introduced the Linear Perfect Phylogeny Flipping problem and showed that it is NP-hard. To solve this problem, we developed a method named Phyolin that takes as input a binary matrix of single-cell DNA sequencing data and then identifies a linear perfect phylogeny in the data by assuming that any implied branching are actually false negatives. The outputted number of flips from Phyolin is then used to compute the strength of the evidence of the two alternatives of the pattern of evolution. We tested Phyolin on both simulated data and real data and showed that it is more accurate than a recent deep learning method [14] and concurs with previously inferred evolutionary patterns on two real datasets. In conclusion, Phyolin is a reliable, easy to use and fast method to assess the likelihood of a linear evolution before more complex reconstruction methods are utilized.

There are several future research directions. First, Phyolin could easily incorporate mutation and cell clustering through additional constraints when supplied with a number of cell clusters and/or mutation clusters. A search could be performed to find the optimal number of clusters such that the likelihood of the data of is maximized. Second, even when the phylogeny is branched, the trunk of the tree may be linear or there might be a long branch with linear evolution within that branch. This means that a subset of the mutations form a linear perfect phylogeny. A future direction is to explore if Phyolin can identify a subset of mutations that are likely to be truncal or form a long branch of the tree, thus potentially providing fast partial inference of the tree.

Finally, exploring evolutionary models that allow ISA violations, such as mutation loss, is an exciting direction for future study. In particular, the 1-Dollo evolutionary, where each mutation is gained exactly once and subsequently lost at most once, could potentially be achieved by replicating each column once and then using Phyolin. If the two columns representing the same mutation are distinct in the inferred linear perfect phylogeny, then that implies that the mutation was lost once. The plausibility of a linear perfect phylogeny under both ISA and under a 1-Dollo model could be compared [3].

## Data Availability

Phyolin is available at https://github.com/elkebir-group/phyolin.

## Footnotes

1 https://www.ibm.com/analytics/cplex-cp-optimizer.
